# Concentrated LiFSI–Ethylene Carbonate Electrolytes and Their Compatibility with High-Capacity and High-Voltage Electrodes

**DOI:** 10.1021/acsaem.1c03096

**Published:** 2022-01-10

**Authors:** Burak Aktekin, Guiomar Hernández, Reza Younesi, Daniel Brandell, Kristina Edström

**Affiliations:** Department of Chemistry − Ångström Laboratory, Uppsala University, Box 538, SE-75121 Uppsala, Sweden

**Keywords:** concentrated electrolytes, LiFSI, EC, NMC, silicon-graphite, Li-metal anode, LNMO

## Abstract

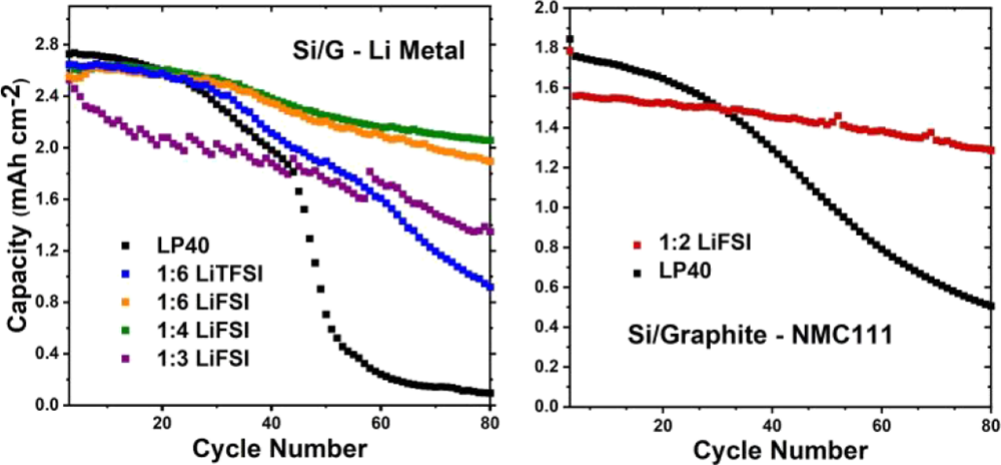

The unusual physical and chemical properties of electrolytes with excessive salt contents have resulted in rising interest in highly concentrated electrolytes, especially for their application in batteries. Here, we report strikingly good electrochemical performance in terms of conductivity and stability for a binary electrolyte system, consisting of lithium bis(fluorosulfonyl)imide (LiFSI) salt and ethylene carbonate (EC) solvent. The electrolyte is explored for different cell configurations spanning both high-capacity and high-voltage electrodes, which are well known for incompatibilities with conventional electrolyte systems: Li metal, Si/graphite composites, LiNi_0.33_Mn_0.33_Co_0.33_O_2_ (NMC111), and LiNi_0.5_Mn_1.5_O_4_ (LNMO). As compared to a LiTFSI counterpart as well as a common LP40 electrolyte, it is seen that the LiFSI:EC electrolyte system is superior in Li-metal–Si/graphite cells. Moreover, in the absence of Li metal, it is possible to use highly concentrated electrolytes (e.g., 1:2 salt:solvent molar ratio), and a considerable improvement on the electrochemical performance of NMC111–Si/graphite cells was achieved with the LiFSI:EC 1:2 electrolyte both at the room temperature and elevated temperature (55 °C). Surface characterization with scanning electron microscopy (SEM) and X-ray photoelectron spectroscopy (XPS) showed the presence of thicker surface film formation with the LiFSI-based electrolyte as compared to the reference electrolyte (LP40) for both positive and negative electrodes, indicating better passivation ability of such surface films during extended cycling. Despite displaying good stability with the NMC111 positive electrode, the LiFSI-based electrolyte showed less compatibility with the high-voltage spinel LNMO electrode (∼4.7 V vs Li^+^/Li).

## Introduction

The development of advanced lithium-ion batteries (LiBs), such as generation 3b in the European strategic energy technology (SET) plan,^1^ is still of the highest priority not only for the fastest-growing energy-storage applications, that is, electric vehicles but also for large-scale storage and many others. The main requirements for an advanced LiB are high energy/power density, long cycle life, high safety, and low environmental impact/cost in their production and usage. Ideally, electrode materials used in a LiB should have high specific-charge capacity and deliver high cell voltage, meaning that the cathode (positive electrode) should have a high and the anode (negative electrode) should have a low operation potential. Unfortunately, this requirement also brings significant (electro)chemical instability issues in conventional LiB electrolyte systems (consisting of LiPF_6_ salt dissolved in organic carbonate solvents) as they possess a rather limited electrochemical stability window.^2^ As a result, undesired electrolyte reduction and oxidation reactions occur at the anode and cathode, respectively, which thus deteriorate the battery cycle life.^3−7^ In the case of anodes with low operating potentials such as Li metal or graphite, Li dendrite formation is another problem as this can cause a short circuit between two electrodes.^8^ Similar problems appear for many high-capacity electrodes such as Si, where the electrolyte instability correlates with large electrode volume expansions, rendering excessive formation of solid electrolyte interphase (SEI) material and depletion of the Li inventory. Also, LiPF_6_ salt has low thermal stability, and carbonate-based solvents are volatile and flammable, making these common electrolyte systems prone to thermal decomposition at temperatures as low as 100–120 °C.^9,10^ Consequently, these electrolyte-related problems bring a negative impact on the battery cycle life and safety.

One promising direction to overcome these hurdles is to increase the Li salt concentration in the electrolyte to sufficiently high levels. The realization of unusual physical and chemical properties of highly concentrated electrolytes (HCEs), or solvent-in-salt electrolytes, has attracted significant interest in recent years,^11,12^ despite HCEs also being associated with higher viscosity, lower ionic conductivity, and higher cost. At sufficiently high salt concentrations, the number of nonsolvating molecules is diminished to a high extent, and the solution structure of Li^+^ in the electrolyte is thus significantly altered. For some specific salt–solvent combinations, this alteration has the potential to improve reductive/oxidative stability, decrease the volatility, and increase the thermal stability, while still enabling high rate cycling of cells.^13^ For instance, stable cycling of graphite anodes has been reported for concentrated propylene carbonate (PC)-^14^ and acetonitrile (AN)^15^-based electrolytes. For the Li metal anode, highly concentrated ether-based electrolytes with lithium bis(fluorosulfonyl)imide (LiFSI) salt can enable dendrite-free plating at high rates with a high Coulombic efficiency (CE).^16^ In the case of the dimethyl carbonate (DMC) solvent, considerably high salt concentrations can be achieved,^11,17^ and such electrolytes have been shown to inhibit transition-metal dissolution and Al current collector corrosion at voltages as high as 4.7 V (vs Li^+^/Li), enabling stable cycling in LiNi_0.5_Mn_1.5_O_4_/graphite full cells.^11^ In other studies, improved oxidation resistance of different HCE systems has been reported, and it was shown that better electrochemical performance can be achieved in full cells or half cells also with layered oxide cathode materials such as LiNi_*x*_Mn_*y*_Co_1–*x*–*y*_O_2_ (NMC).^18−26^

Ethylene carbonate (EC) is a common solvent used in LiB electrolytes. It has a rather high melting temperature around 36 °C and is therefore usually mixed with other solvents with low melting temperatures, for example, diethyl carbonate (DEC), DMC, or ethyl methyl carbonate (EMC).^8^ Even though EC is known to oxidize at high voltages (>4.2 V vs Li^+^/Li),^27^ it has been a vital component of conventional electrolytes because of its robust SEI-forming ability on the graphite anode surface.^28^ McOwen et al.^29^ reported that the HCE approach can improve the thermal and high voltage stability in a LiPF_6_-free electrolyte consisting of pure EC solvent and bis(trifluoromethane)sulfonimide lithium salt (LiTFSI). The concentrated electrolyte (e.g., 1:3 molar ratio of LiTFSI:EC) remained liquid at room temperature and suppressed the corrosion of the Al current collector.^29^ Later, Nilsson et al.^30^ studied different concentrations of the same electrolyte system in a variety of Li-metal-based cells such as Li-Li symmetrical cells and “anode-free” LiFePO_4_ (LFP) cells and showed that LiTFSI:EC-based HCEs hold promise of stable and safe electrochemical cycling of Li metal cells at reasonable current rates.^30^

In this study, we adopt a similar HCE approach with pure EC solvent; however, we use LiFSI salt instead of LiTFSI. In earlier studies with LiTFSI, the ionic conductivity of the highly concentrated electrolyte, for example, LiTFSI:EC ratios of 1:3 and 1:2, has been rather low at room temperature (<1 mS cm^–1^).^29^ Therefore, relatively low salt concentrations such as the LiTFSI:EC ratio of 1:6 had to be employed in electrochemical testing because the low ionic conductivity, high viscosity, and poor wettability of the electrolyte resulted in quite high cell resistances at higher concentrations.^30^ However, in the case of higher voltage cathodes (as compared to LFP), the use of HCEs with “relatively low” salt concentrations will most likely pose severe stability issues and generate electrolyte oxidation and Al current collector corrosion. Therefore, there is a need to develop well-functioning salt–solvent systems for HCEs.

In this work, we show that the choice of the smaller and lighter FSI anion over TFSI^–^ extends the upper voltage limit of the electrolyte stability window with a less negative effect on ionic conductivity. Highly concentrated LiFSI:EC electrolytes improve the electrochemical cycling stability of NMC111, silicon/graphite, and Li metal electrode-based full and half cells, without the need for additional electrolyte additives.

## Experimental Section

### Materials and Electrolyte

Commercial LiNi_0.44_Mn_1.56_O_4_ (LNMO) powders^31^ were used to prepare composite electrodes comprising 92 wt % active material, 4 wt % carbon black (Imerys, C65), and 4 wt % poly(vinylidene difluoride) based binder (PVdF-HFP; Kynar Flex 2801). Composite electrode sheets of NMC111 and Si-graphite were bought from Customcells Itzehoe GmbH (Germany). Si-graphite electrodes comprised 77.5 wt % graphite, 12.5 wt % Si, and 10 wt % nonactive components such as conductive carbon and carboxymethyl cellulose (CMC) - styrene butadiene rubber (SBR) based binder. In half cells, Li metal from Cyprus Foote Minerals was used (with 2.6 cm diameter and 125 μm thickness). The separators used in cells were Solupor 3P07A (Lydall, single layer polyethylene, 20 μm) and Whatman glass fiber (GE Life Sciences, 240 μm). Electrodes and separators were vacuum-dried for 10 h at 100 and 70 °C, respectively, inside a glovebox (Ar atmosphere, H_2_O < 5 ppm, and O_2_ < 1 ppm). LiTFSI powders (BASF) were dried similarly at 120 °C for 24 h and LiFSI powders (Suzhou Fluolyte Co.) at 90 °C for 24 h. Stoichiometric amounts of Li salts and the EC solvent (Gotion Inc.) were mixed by magnetic stirring at 70 °C for 24 h. The electrolytes with different concentrations are denoted as salt:solvent names, followed by their molar ratios, for example, “LiFSI:EC 1:2”. All electrolyte preparation took place in the same glovebox. The LP40 electrolyte, that is, 1 M LiPF_6_ in 1:1 (vol.) EC:DEC, was used as a reference electrolyte for comparison purposes (Gotion Inc.). The molar ratios of components in the LP40 electrolyte are “LiPF_6_:EC:DEC 1:7.1:3.9” corresponding to a salt:solvent ratio of 1:11. Electrolyte conductivities were measured using a Mettler Toledo SevenGo duo pro SG78 meter connected to InLab 738ISM sensors. These measurements were carried out in the same glovebox in which electrolyte preparation took place. LiFSI salt used in the conductivity measurements was bought from Provisco CS (purity: 98.0%). For one chosen composition, LiFSI:EC 1:3, LiFSI from both Provisco CS and Suzhou Fluolyte were tested to ensure that the salts from different suppliers do not affect the ionic conductivity of electrolytes. Differential scanning calorimetry (DSC) experiments were performed with a Mettler Toledo DSC3+. Approximately 8 mg of sample was placed in a hermetically sealed aluminum crucible. The samples were cooled to −60 °C at a rate of 1 °C min^–1^, held at −60 °C for 5 min, and heated until 80 °C at 1 °C min^–1^ rate. Dynamic viscosity of the electrolytes was measured with a LOVIS 200 ME microviscosimeter module connected to a DMA 4100 M density meter (Anton Paar). The measurements were performed from 70 to 10 °C in 10 °C steps.

### Electrochemical Testing

Mass loadings of LNMO electrodes were in the range of 12 mg cm^–2^, corresponding to a theoretical capacity around 1.6 mAh cm^–2^. Electrodes were punched to 2 cm diameter discs and then calendared under high pressure (∼160 MPa). The nominal capacities of NMC111 and Si-graphite electrodes were 2 and 2.2 mAh cm^–2^, respectively. Thus, the full cells were cathode-limited. These electrodes were likewise punched to 2 cm diameter discs. In half cells, the Li metal electrode diameter was 2.6 cm. The electrolyte amount was 120 μL in full cells and 240 μL in half cells. In full cells, two layers of Solupor separator, and in half cells, one layer of glass fiber + one layer of Solupor (facing the Li side) separator were used. All cells were in the pouch cell format, as described in detail in earlier work.^7^ In the linear sweep voltammetry (LSV) experiment, Al foil was used as the working electrode and Li metal as the counter and reference electrodes. The instrument used was an MPG-2 (Biologic), and the scan rate was 10 mV/min (∼0.17 mV/s). Room-temperature galvanostatic cycling of full and half cells was carried out using a Neware BTS4000 battery testing instrument. After an initial 10 h open-circuit voltage (OCV) step, the first three cycles were performed at 0.2 mA cm^–2^ and the following cycles at 1 mA cm^–2^ current. In full cells, unless specified, constant voltage (CV) steps were not added at the end of constant current charging/discharging steps (and only applied at the end of charging when specified). The upper and lower cutoff voltage limits were 4.2 and 3.0 V, respectively. In Si-graphite/Li half cells, a CV step was added at the end of discharge during 1 mA cm^–2^ cycling (applied until the current dropped below 0.2 mA cm^–2^). In these cells, the upper and lower cutoff voltage limits were 1 and 0.01 V vs Li^+^/Li, respectively. LNMO-based cells were also tested to check the compatibility of electrolytes at significantly higher voltages (cycled between 3.5 and 4.95 V vs Li^+^/Li). In order to compare the degree of side reactions between the concentrated and reference electrolytes, low-rate cycling (0.2 mA cm^–2^) of NMC and Si-graphite-based half cells and full cells were performed at 55 °C using a high-precision (Novonix HPC) battery tester.

### Ex Situ Characterization of Electrodes

Following the electrochemical cycling, samples were opened in an argon glovebox. Electrodes were washed by 4–5 droplets of DMC to remove any electrolyte residues, and this step was repeated three times. For the morphological analysis, electrodes (part of the 2 cm diameter electrodes) were transferred into a scanning electron microscope (Merlin, Carl Zeiss, Germany) equipped with a field emission gun. Transfer was performed using an airtight transfer vessel without exposing the samples to air. Imaging was performed at an operation voltage of 3 kV and a beam current of 100 pA. Elemental analysis was also performed with the same instrument by energy-dispersive X-ray spectroscopy (EDS). The second set of samples was prepared from the remaining part of the electrodes and were similarly transferred into a Phi-5500 X-ray photoelectron spectroscopy (XPS) instrument for surface analysis. XPS analysis was carried out using monochromatic Al-Kα radiation (1486.6 eV). No charge compensation was applied during the measurements. Casa XPS software was used for the data analysis. For all spectra, a linear energy calibration was applied so that the hydrocarbon peak is positioned at 285 eV. Data are presented following Shirley background subtraction and subsequent normalization for each element spectra (intensity divided by the maximum value). When fitting, Gaussian/Lorentzian peak shape GL(30) was used for all peaks, except the conductive carbon peak, which was fitted to a GL(80) peak shape.

## Results and Discussion

### Ionic Conductivity and High Voltage Stability

**Figure 1:**
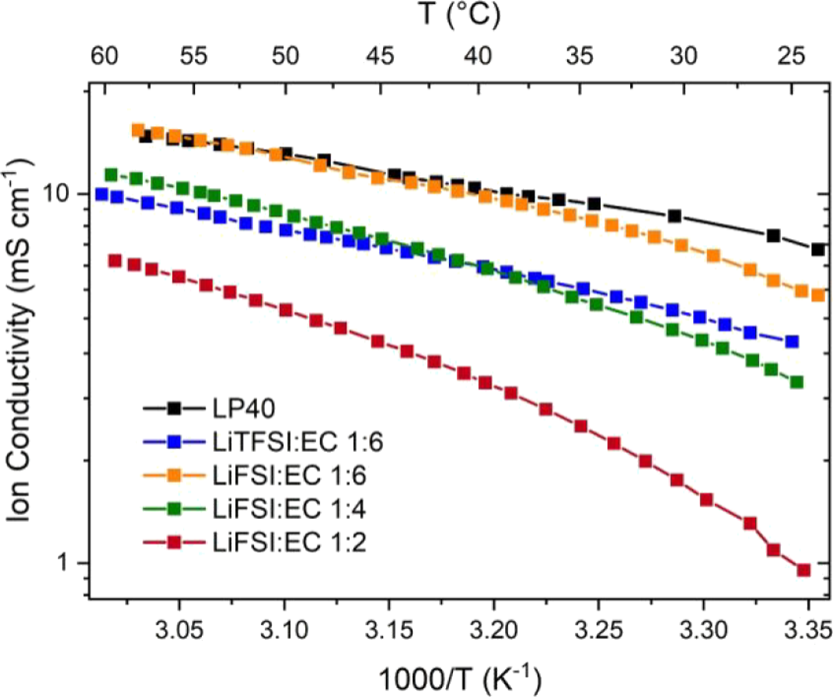
Ionic conductivities of LiFSI:EC electrolytes at different salt concentrations. LP40 and LiTFSI:EC 1:6 electrolytes were also measured for reference purposes.

LiFSI:EC electrolytes were prepared at different concentrations ranging between 1:6 and 1:2 molar ratios. As expected, increasing the salt concentration resulted in a more viscous liquid solution (see Figure S1 for viscosity measurements). While no salt residues were observed in the electrolyte in the studied concentration range, some negligible number of solid residues could be spotted after several days at the bottom of the glass vial containing the LiFSI:EC 1:2 electrolyte. The viscosity was also noticeably higher as compared to other concentrations. Therefore, concentrations higher than LiFSI:EC 1:2 were not prepared. In Figure 1, ionic conductivities of LiFSI:EC electrolytes with different salt concentrations are shown together with the reference electrolytes, that is, LP40 and LiTFSI:EC 1:6 electrolytes.

**Figure 2:**
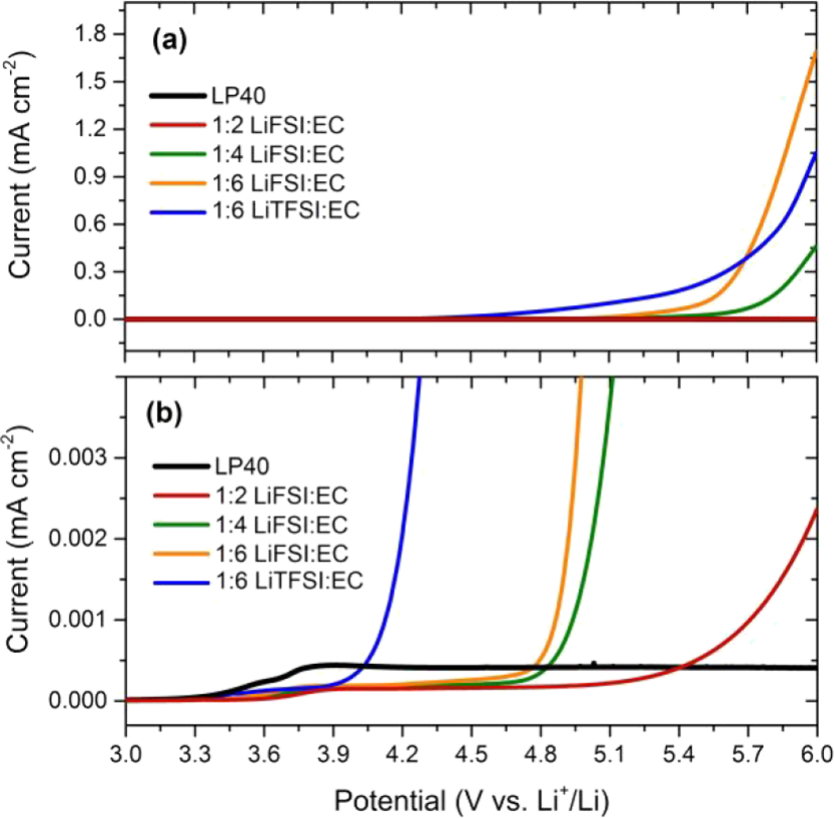
High voltage stability of different electrolytes as measured with LSV in Al-Li cell configurations at room temperature. The same data are presented with different y-axis scales in (a) and (b) for easier interpretation. The scan rate is 10 mV/min (∼0.17 mV/s).

In the temperature range of ∼25 to 60 °C, the LP40 reference electrolyte (1:11 salt:solvent molar ratio), as expected, has the highest conductivity. Strikingly, however, the LiFSI:EC 1:6 electrolyte displays similar conductivities, especially at temperatures above 35 °C. For the same salt:solvent ratio of 1:6, the LiFSI-based electrolyte has considerably higher conductivity as compared to its LiTFSI counterpart, which also has a higher molecular weight. In an earlier study,^30^ the ionic conductivity of the LiTFSI:EC electrolyte at 30 °C was reported to decrease from 3 mS cm^–1^ at 1:6 molar ratio to 0.2 mS cm^–1^ at 1:2 ratio. Here, we measured the conductivity of the LiTFSI:EC 1:6 electrolyte as 4.6 mS cm^–1^ at 30 °C. Upon the substitution of LiTFSI with LiFSI, the conductivity increased to 7 mS cm^–1^ (1:6 molar ratio), while the viscosity of the LiFSI-based electrolyte was slightly lower (16.8 mPa s for LiFSI:EC 1:6 vs 19.4 mPa s for LiTFSI:EC 1:6). The conductivity decreased to only 1.5 mS cm^–1^ when the LiFSI concentration was increased to 1:2. This is more than a magnitude higher compared to its LiTFSI counterpart. This is a promising result because it shows that the increasing molar salt concentration has a significantly less severe impact on ionic conductivity for the LiFSI salt than for LiTFSI. The LiFSI:EC 1:2 electrolyte remained liquid in the temperature range of −60 to 80 °C (see DSC results in Figure S1). Similarly, the LiFSI:EC 1:6 electrolyte also remained liquid, while the LiFSI:EC 1:4 electrolyte showed a melting point close to −20 °C. This indicates the presence of a congruent melting point near the 1:4 concentration in the phase diagram. At this point, it is also important to investigate the impact of salt concentration on the side reactions occurring at high voltages (e.g., on the Al current collector). For this purpose, LSV tests were performed at a 10 mV/min (∼0.17 mV/s) scan rate in Al-Li cell configuration, and the results are shown in Figure 2.

The corrosive decomposition reaction of the LiTFSI-based electrolyte with the Al working electrode starts around 3.9–4.0 V (vs Li^+^/Li), in agreement with the literature.^29^ As seen in Figure 2b, for the same salt:solvent ratio of 1:6, the LiFSI-based electrolyte shows a higher stability than the LiTFSI-based counterpart. The onset of side reactions is observed at around 4.7–4.8 V (vs Li^+^/Li) for the LiFSI-based electrolyte (0.8 V higher than the LiTFSI-based electrolyte). At a higher LiFSI:EC concentration of 1:4, side reactions are further suppressed, especially at high voltages (see Figure 2a). When the concentration is increased further (LiFSI:EC 1:2), neither Al corrosion nor electrolyte oxidation starts until 5.2–5.4 V (vs Li^+^/Li). These observations are likely the result of reduced solvent interactions with the Al metal surface and low metal dissolution rate predictable at high salt concentrations.^29^ Because the degree of side reactions on Al metal can get worse during subsequent cycling (or at prolonged times), the stability of the electrolyte over extended LSV cycles was also tested. As shown in Figure S2, the best-performing electrolyte (LiFSI:EC 1:2) was tested for 50 cycles between 3.0 and 5.0 V (vs Li^+^/Li), and it was confirmed that the passivation of the Al current collector was persistent also over extended cycling. These results indicate that LiFSI:EC-based electrolytes can be high-voltage-compatible even without the presence of electrolyte additives. Therefore, the compatibility of this electrolyte system with the NMC and LNMO cathodes is also worth investigating; however, it is sensible to focus first on the electrochemical performance with “low voltage” but high-capacity anodes (e.g., Li metal and Si-graphite anodes), which is likewise of crucial importance in a practical high-voltage battery.

### Si/Graphite–Li Metal Half Cells

**Figure 3:**
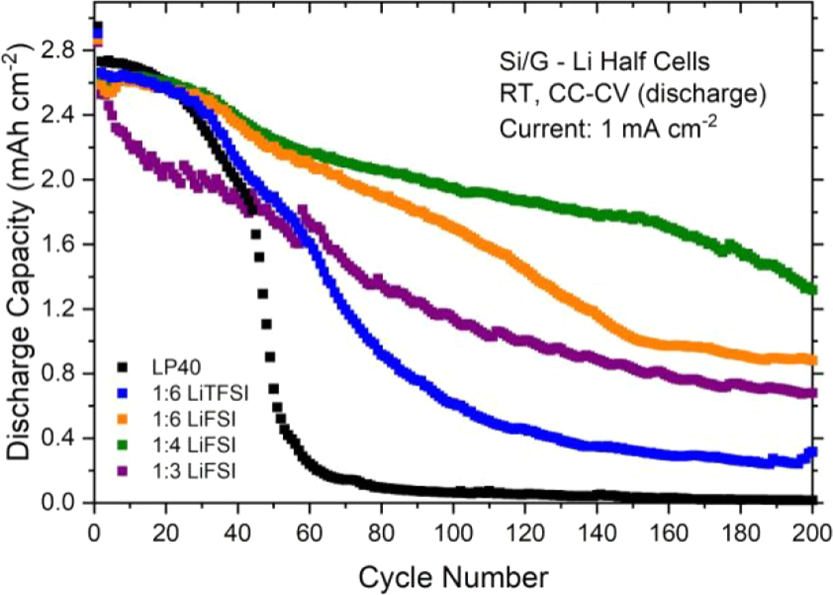
Electrochemical cycling results of Si/graphite–Li half cells. The current is 1 mA cm^–2^ during charge/discharge, and the CV voltage step (10 mV) is only applied at the end of discharge until the current reaches 0.2 mA cm^–2^.

Concentrated LiTFSI- and LiFSI-based electrolytes are well known for their beneficial effect on the cycling stability of Li metal-based batteries.^16,19,26,32−36^ Nilsson et al.^30^ reported that a LiTFSI:EC 1:6 electrolyte was superior to reference electrolytes when cycled in symmetrical Li-Li cells and anode-free Li-Cu cells. Here, the electrolytes are tested in Si/graphite-based cells with a Li counter electrode. Si/graphite electrodes also operate at voltages near Li metal, and it is thus possible to investigate the low-voltage performance with respect to stability against side reactions (Si/graphite) as well as the Li plating/stripping performance (Li metal). The cycling results of such cells with different electrolytes are shown in Figure 3.

All cells initially deliver a high discharge capacity around 2.6–2.7 mAh cm^–2^ during the early stages of cycling. Both the charge and discharge currents are 1 mA cm^–2^, and considering that the capacity for each cycle is also reasonably high, it can be expected that stripping/plating on Li metal will contribute significantly to impedance growth and thus to cell failure. Therefore, these results should not be compared in a direct manner with the literature in which cell testing is performed with rather low mass loading electrodes and thus low current per area. This is also the reason why we here report the actual cell currents and capacities rather than the C-rates and specific capacities. Under these testing conditions, it is seen that the LiPF_6_-based reference electrolyte (LP40 electrolyte without any additives) shows a cell failure (∼50% capacity loss) already after 50 cycles. An improvement in cycling performance is observed with the concentrated LiTFSI-based electrolyte (LiTFSI:EC 1:6); however, this improvement is limited, and the cell failure is delayed to merely 70 cycles. This observation for the LiTFSI:EC 1:6 electrolyte is in agreement with an earlier cycling data reported for LFP-Li half cells cycled under similar conditions.^30^ It should be noted that the contribution of the LFP cathode to the cell failure should be limited in that cell configuration because of its stability and low cathode voltage. This indicates that the contribution of Li metal to cell failure is more significant than that of the Si/graphite electrode under these cycling conditions (e.g., capacity >2 mAh cm^–2^, current >1 mA cm^–2^).

At the same salt:solvent ratio, a more substantial improvement in cell performance is observed for the LiFSI salt, and the cell failure (∼50% capacity loss) is further delayed to 130 cycles. When the salt concentration is increased to LiFSI:EC 1:4, cell failure is not observed until 200 cycles. However, further increase in the salt concentration exhibited adverse effects on the performance. First, LiFSI:EC 1:2 electrolytes were tested (not shown here); however, unexpectedly high charge capacities were observed when the current was increased to 1 mA cm^–2^ from 0.2 cm^–2^. The voltage profile also showed an irregular behavior with voltage spikes, indicative of a problematic Li plating process. This can be due to the inferior wettability properties and high viscosity (see Figure S1) of the highly concentrated electrolyte, which can affect the current distribution at the Li metal surface. This indicates that there is a positive effect of increasing salt concentration on SEI formation and/or Li plating/stripping behavior, but this effect seems to be overcome by the negative effect of poor wettability and ionic conductivity at very high concentrations. Thereafter, we investigated the performance of a concentration between 1:2 and 1:4, that is, LiFSI:EC 1:3 electrolyte. The ionic conductivity (see Figure S3) of this electrolyte was intermediate between the 1:2 and 1:4 electrolytes, and the wetting of separators was less problematic as compared to the 1:2 electrolyte. As seen in Figure 3, it was possible to cycle the cell prepared with the 1:3 concentration, but the performance was still worse as compared to the 1:4 electrolyte. The difference in discharge capacities became evident as soon as the current was increased to 1 mA cm^–2^ from 0.2 cm^–2^ (see also Figure S4).

These results clearly show that the positive effect of increasing salt concentration is overcome by the negative effect of poor wettability and ionic conductivity after a certain threshold of salt concentration. In preliminary experiments, the wettability of the electrolyte with the separator was observed to be problematic with Celgard separators, and better results were obtained with glass fiber and Solupor separators. It is therefore likely that the separator–electrolyte and Li-electrolyte wettability plays an important role in the cycling performance. Based on our observations, the problems at concentrations above 1:4 seem to originate mainly during the charging step (during Li plating), and it might, therefore, still be possible to use higher concentration electrolytes in different cell configurations where the electrolyte wettability would not be as critical as for Li-metal based cells. To this end, we tested the highly concentrated LiFSI:EC 1:2 electrolyte in full cells using NMC111 and Si/graphite electrodes.

### NMC111–Si/Graphite Full Cells

**Figure 4:**
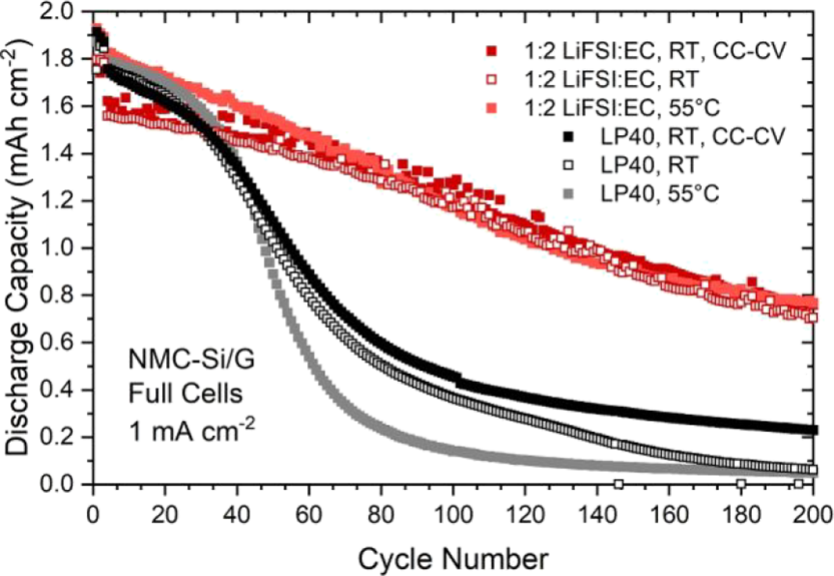
Electrochemical cycling results of NMC111–Si/graphite full cells. The nominal capacity of the NCM111 electrodes is 2 mAh cm^–2^.

In this cell configuration, the upper cell voltage is 4.2 V vs Li^+^/Li. Thereby, the stability of LiFSI:EC-based concentrated electrolyte at higher voltages is tested. In addition, this is a full cell configuration, and the amount of Li inventory is limited and determined by the initial capacity of the NMC111 electrode (∼2 mAh cm^–2^). Therefore, it will also be possible to see the effect of side reactions on the Li inventory loss. As the LSV tests in Figure 2 show, the highest oxidation stability was achieved for the 1:2 concentration. For this reason, as being the best candidate in this respect, we tested this electrolyte under different cycling conditions together with the LP40 electrolyte as a reference (see Figure 4).

The electrochemical testing of the LiFSI:EC 1:2 electrolyte using the same constant current (CC)-CV cycling conditions as for the Si/G–Li half cells did not cause similar problems in full cells. This shows that the wettability and ionic conductivity become a major problem primarily in the presence of a Li metal electrode. The cycling stability was also studied without the CV steps. As seen in Figure 4, the CC cycling results are not very different from the CC-CV cycling results, showing that the overpotential during charging (i.e., lithiation of Si/graphite) is not the most significant factor determining the capacity fading during cycling. When the temperature is increased to 55 °C, the initial performance of the LP40-based cells remains the same, but in later periods of cycling, this cell fades more quickly. This can be related to a higher temperature sensitivity of the LiPF_6_ salt and associated decomposition reactions (e.g., HF generation, cathode side reactions, and so forth.) during cell operation, and to SEI dissolution (i.e., side reactions on the Si/graphite). In the case of the LiFSI-based electrolyte, there is some increase in discharge capacity because the ionic conductivity increases from ∼1 mS cm^–1^ at 25 °C to 6 mS cm^–1^ at 55 °C. In the long term, however, the performance of all LiFSI-based cells is comparable. These results show that the LiFSI:EC 1:2 electrolyte is quite suitable for the cycling of high-voltage NCM111 electrode-based full cells. In order to better understand the reasons for the performance difference, it is required to perform further electrochemical testing with a focus on side reactions and Coulombic efficiency (CE) because the CE values are quite dependent on the applied current and prone to deviations between subsequent cycles. This is particularly more important in this cell chemistry because of the sensitivity of electrode potentials to the upper cutoff voltage, Li-trapping, and Li-plating effects (Si/graphite) and low voltage kinetic hindrance behavior of the NMC electrode, especially at high currents and with the initial formation cycles. For these reasons, identical cell configurations were tested under specific cycling conditions using a high-precision charger system.

### CE Measurements

**Figure 5:**
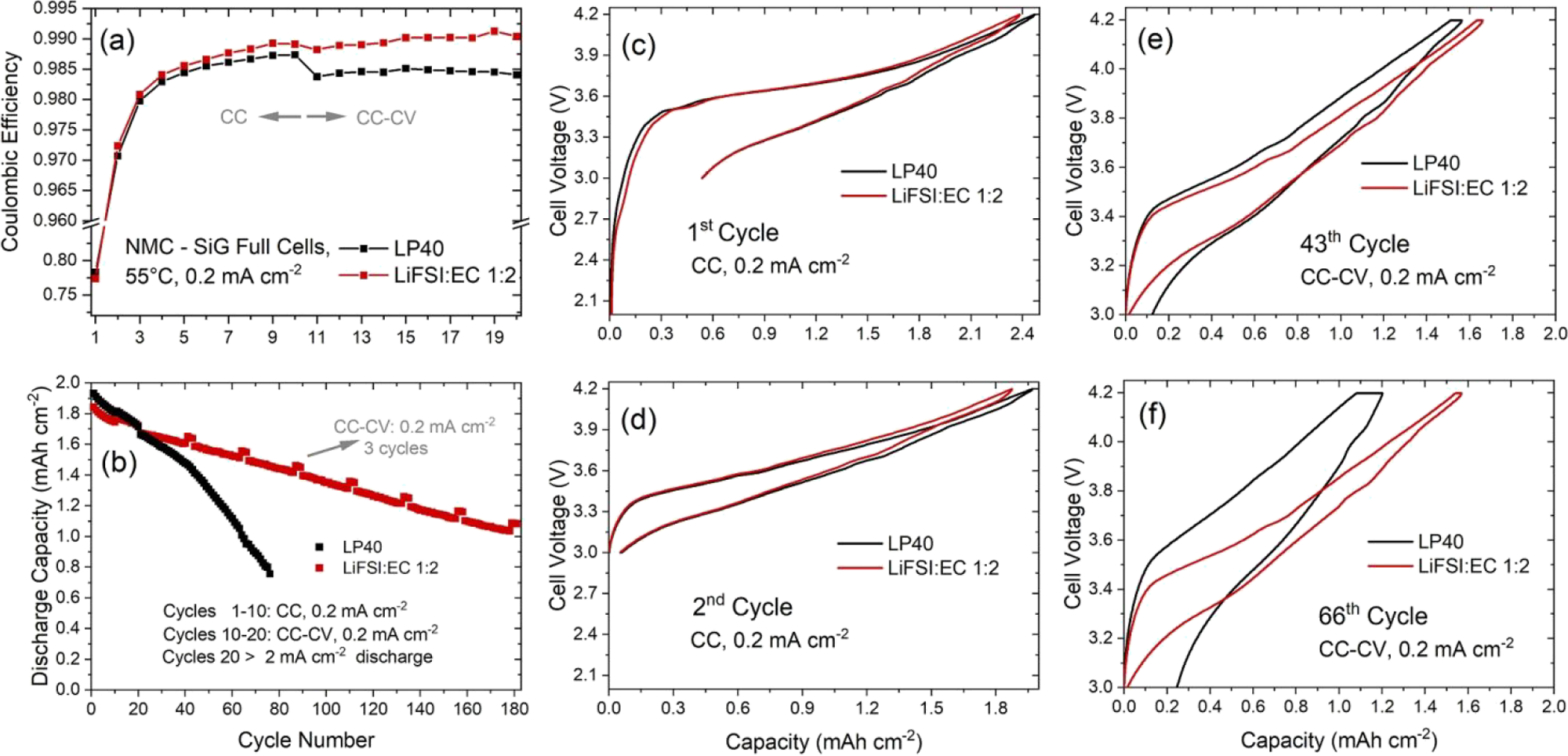
Electrochemical cycling results of NMC111–Si/graphite full cells cycled with a high-precision charger at 55 °C. Both charge and discharge currents are 0.2 mA cm^–2^ during the first 10 cycles. Potentiostatic holding and OCV steps were applied at the end of the charging step during cycles 10–20, and Coulombic efficiency results are shown in (a). In the following cycles, the discharge rate was increased to 2 mA cm^–2^. After every 20 cycles, 3 cycles with 0.2 mA cm^–2^ were applied. The results of the overall test are shown in (b). Voltage profiles from selected cycles are shown in (c–f).

The aim of the CE tests is to observe the degree of side reactions occurring for the reference and LiFSI-EC-based electrolytes. Kinetic limitations during charging and discharging can affect the CE values, and we therefore performed the tests at low currents to minimize the effect of kinetic factors on measured CE values. For the same reason, cell testing was performed at 55 °C because side reactions can be expected to be more severe, while kinetic effects are less pronounced at this temperature (see Figure 5). During the first 10 cycles, both charge and discharge currents were rather low, that is, 0.2 mA cm^–2^. In the subsequent 10 cycles, potentiostatic and OCV steps were applied at the end of charging to observe the effect of the electrolyte on the self-discharge rate in the fully charged state. After 20 cycles, the same cells were further cycled in an asymmetric way with a higher discharge rate at 2 mA cm^–2^ with the aim to see the effect of side reactions also on the discharge kinetics.

The first cycle CE values (see Figure 5a) are similar for both cells with the LiFSI-EC electrolyte displaying a slightly lower value. The Si/graphite electrode is expected to consume more charge because of side reactions, and the CE value of the full cell should therefore be determined by the degree of electrolyte side (reduction) reactions on the anode if the kinetic effects are neglected.^7^ However, NMC-type cathodes are known to exhibit a low voltage kinetic hindrance during discharging, and this has an effect on the first cycle discharge capacity even at low cycling rates.^37^ Nevertheless, voltage profiles in Figure 5c show that the difference between the two electrolytes mainly originates during the charging step. The LiFSI-based electrolyte displays an additional small plateau around 2.7–2.9 V, and at the end of charging, the LP40-based cell delivers a larger capacity. This indicates that the small plateau around 2.7–2.9 V might be an indirect result (e.g., change in the cell voltage) of side reactions on the Si/graphite electrode.

It should be noted that the electrolyte oxidation reactions on the cathode would increase the capacity observed during charging, and in order to investigate the electrolyte oxidation reactions further, it is useful to test the same NMC111 electrodes also in half cells because the Li counter electrode can provide (or host) excess amounts of Li which can be consumed (or gained) during electrolyte reduction (or oxidation) reactions. This would enable seeing the degree of side reactions on the cathode only, as side reactions on the Li metal anode would not be reflected in the CE values (as long as the effects of kinetics are negligible). The testing of such half cells (see Figure S5) showed that the CE values are slightly lower for the LiFSI-based electrolyte in the first three cycles. This indicates that the extra charge for the LP40 electrolyte is not caused by additional side reactions. On the other hand, as expected for both electrolytes, the CE values in NMC111 half cells are higher compared to full cells, confirming that the side reactions on the anode side are more severe and thus determine the overall CE of the full cells (including the first cycle).

In full cells, already after a few cycles, the LiFSI-based electrolyte quickly achieves higher CE values and behaves slightly better than the LP40 electrolyte, as seen in Figure 5a. Similar trends are also seen for the NMC half cells (see Figure S5). This difference becomes even more pronounced when the CV and OCV steps are added. In the charged state, side reactions can occur in a more severe way^38,39^ and cause self-discharge of the electrodes, which would further decrease the CE values.^40^ After introducing the high-voltage CV and OCV steps, a significant drop in CE is observed for the LP40 electrolyte. However, such a drop is not observed for the LiFSI-based electrolyte. This shows that the stability against self-discharge of this electrolyte remains unchanged during static holding in the charged state, highly important for practical battery applications. As a result of lower CE values, the LP40-based cell fades more quickly during cycle number 10–20 even at low cycling rates, indicating a faster rate of cyclable lithium loss on the anode (e.g., more severe electrolyte reduction reactions). However, better stability of the LiFSI electrolyte on the cathode can still contribute to an improved anode performance because oxidation products and dissolved transition metals can otherwise migrate to the anode and cause further side reactions.^7^ As the cycling continues, the difference in discharge capacity becomes more distinct (e.g., after 30 cycles). The individual voltage profiles from the 43rd and 66th cycle (Figure 5e,f) show that the capacity contribution during the potentiostatic step as well as the voltage hysteresis increases significantly for the LP40-based cell. This indicates that the capacity fading gradually starts to become more kinetic-dominated as the cycling continues and causes failure of the LP40-based full cell.

In summary, electrochemical testing of these different cell configurations show that the LiFSI:EC electrolytes enable more stable cycling of Si/graphite–Li metal cells; however, there is an optimum concentration of the salt as too high concentrations cause problems during Li plating/stripping. The LiFSI:EC 1:4 concentration rendered the best performance and enabled a more stable cycling compared to other concentrations and reference electrolytes. In the absence of Li metal (NMC-Si/Graphite full cells), it was possible to use more concentrated electrolytes, for example, LiFSI:EC 1:2, and those cells outperformed the cells with the LP40 electrolyte under varying cycling conditions. At this point, a new concentration with a lower salt content (LiFSI:EC 1:3) was also tested (see Figure S6), and a similar passivation capability was achieved. The results showed that concentrated LiFSI:EC electrolytes can be more reductively/oxidatively stable as compared to the LiPF_6_-based LP40 electrolyte. This is a promising observation because it shows that the LiFSI:EC electrolyte combination is not only beneficial for the anode side but also improves the passivation characteristics on the cathode side. In the light of these results, full cells with a higher voltage cathode were tested using LiNi_0.5_Mn_1.5_O_4_ electrodes, having a standard operation voltage near 4.7 V vs Li^+^/Li (see Figure S7). At this high voltage, the LiFSI:EC 1:2 displayed a gradually increasing overpotential and inferior cell performance after 10–15 cycles, even though the difference in Coulombic efficiency was small as compared to the reference electrolyte (LP40). The reasons for this performance difference were not investigated further, but may be due to cell resistance increase caused by the deposition of electrolyte decomposition products on different cell components^7^ or the loss of contact between the Al current collector and the LNMO particles.^41^ The results in Figure S7 indicate that the compatibility of the pure LiFSI:EC 1:2 electrolyte with the high-voltage cathodes has a limit. On the other hand, this voltage limit can possibly be extended by the addition of small amounts of electrolyte additives.^42^

### Ex Situ Characterization of Electrodes

**Figure 6:**
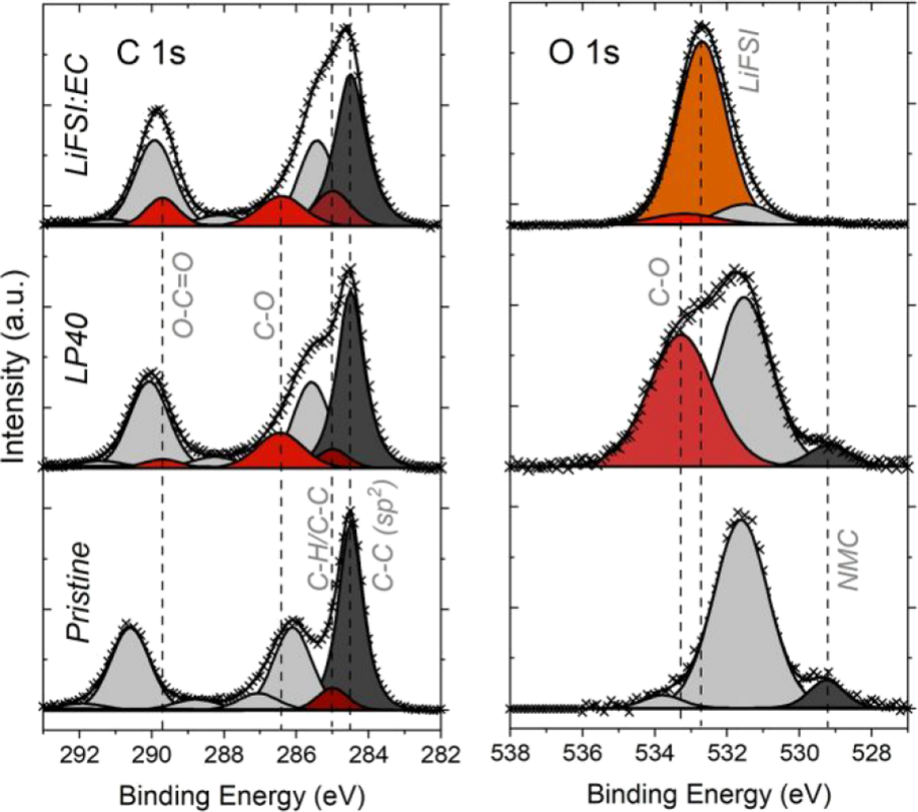
Normalized C 1s and O 1s XPS spectra of NMC111 electrodes before cycling (pristine) and after 10 cycles (using LP40 or 1:2 LiFSI:EC electrolytes).

Surface characterization of NMC111 and Si/graphite electrodes was performed via scanning electron microscopy (SEM)/EDS and XPS after cycling in full cells using LP40 and 1:2 LiFSI:EC electrolytes (10 cycles, 0.2 mA cm^–2^ charge/discharge current). It is known that side reactions occurring at high voltages do not cause a stable layer formation on the surface of cathodes (and conductive carbon additives) when conventional electrolytes are used (e.g., LP40), and such films are known to be relatively thinner as compared to SEI formation on anode surfaces.^7,43^ In the case of NMC111 electrodes, the observation of a significant surface film formation is not expected using the LP40 reference electrolyte. This is confirmed in the SEM analysis (see images in Figure S8) but is also the case for the LiFSI:EC electrolyte. It can thus be concluded that spontaneous surface films resulting from either electrolyte are insufficiently thick for detection by SEM imaging. The same electrodes were also analyzed via XPS for a more surface-sensitive analysis, and the results are shown in Figures 6 and S9.

In the C 1s spectra of the pristine sample, the main peak at 284.5 eV is assigned to the carbon conductive additive in the composite electrode (a 0.5 eV difference between this peak and the hydrocarbon peak was fixed). The other peaks (shown in light gray) mainly originate from the binder components, and their relative positions/areas are fixed in the spectra of cycled electrodes. No specific peak assignments are made for these peaks because commercial electrodes were used. After cycling, binder-related peaks shift to lower binding energies, similar to results reported earlier,^44−46^ and the relative intensity of the conductive carbon peak (dark gray) decreases slightly. This shows that the deposition of side reaction products occurs, but to a limited extent, on the conductive carbon network. The appearance of C–O-related species at around 286.5 eV is observed to a similar extent for both of the cycled electrodes. There is also a smaller contribution from a new peak observed at around 289.5 eV, which can be assigned to O–C=O-related species. This has a stronger contribution for the electrode cycled with the LiFSI:EC electrolyte.

In O 1 s spectra, the metal oxide peak corresponding to the NMC111 particles is seen at 529.4 eV. The relative intensity of this peak decreases after cycling, however, this decrease is more significant in the case of the LiFSI:EC electrolyte, which indicates that the surface films on the cathode particles are thicker for this electrolyte. As expected, both electrodes show C–O-related peaks, but the electrode used with the LiFSI:EC electrolyte shows a larger contribution to the spectra with an intense peak located at around 532.7 eV. This peak is assigned to LiFSI or LiFSI-related species,^19,47^ and the elemental concentration of O and S supports this assumption (see Figure S9c).

**Figure 7:**
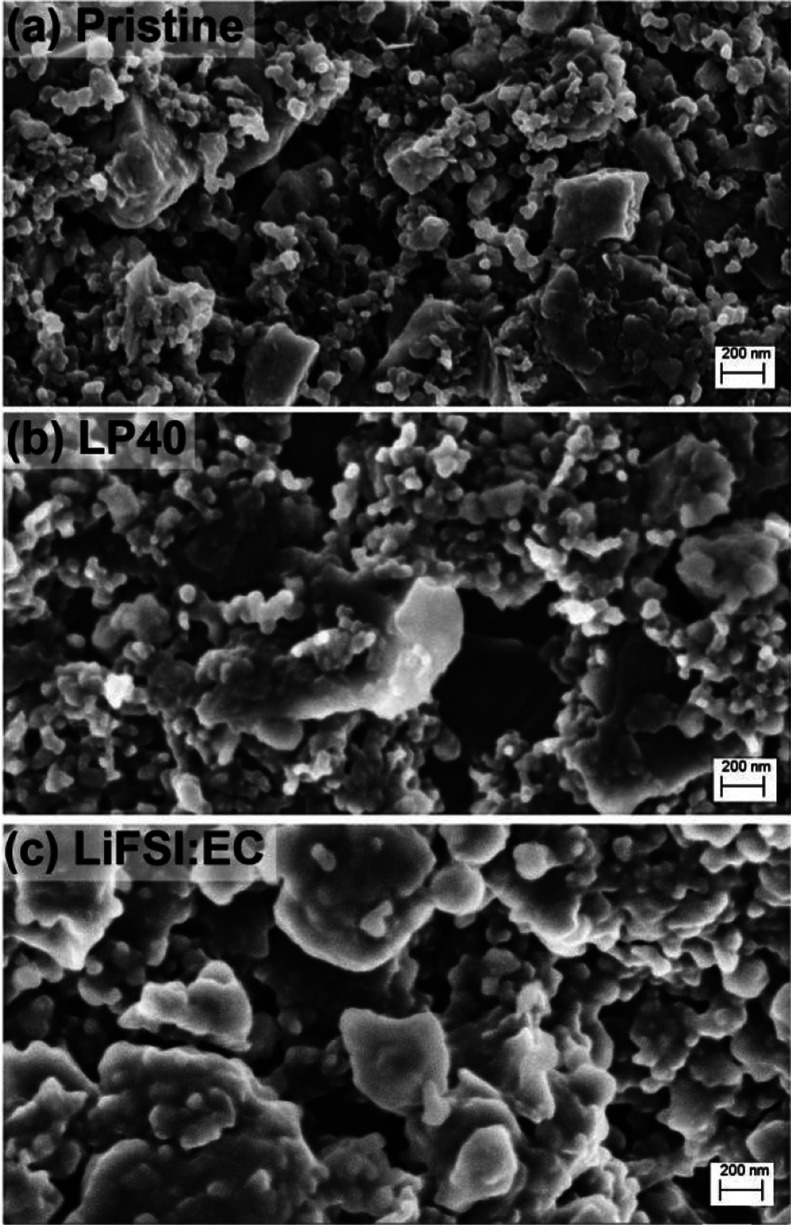
SEM images of Si/graphite electrodes (a) before cycling (pristine) and after 10 cycles using (b) LP40 or (c) 1:2 LiFSI:EC electrolyte.

**Figure 8:**
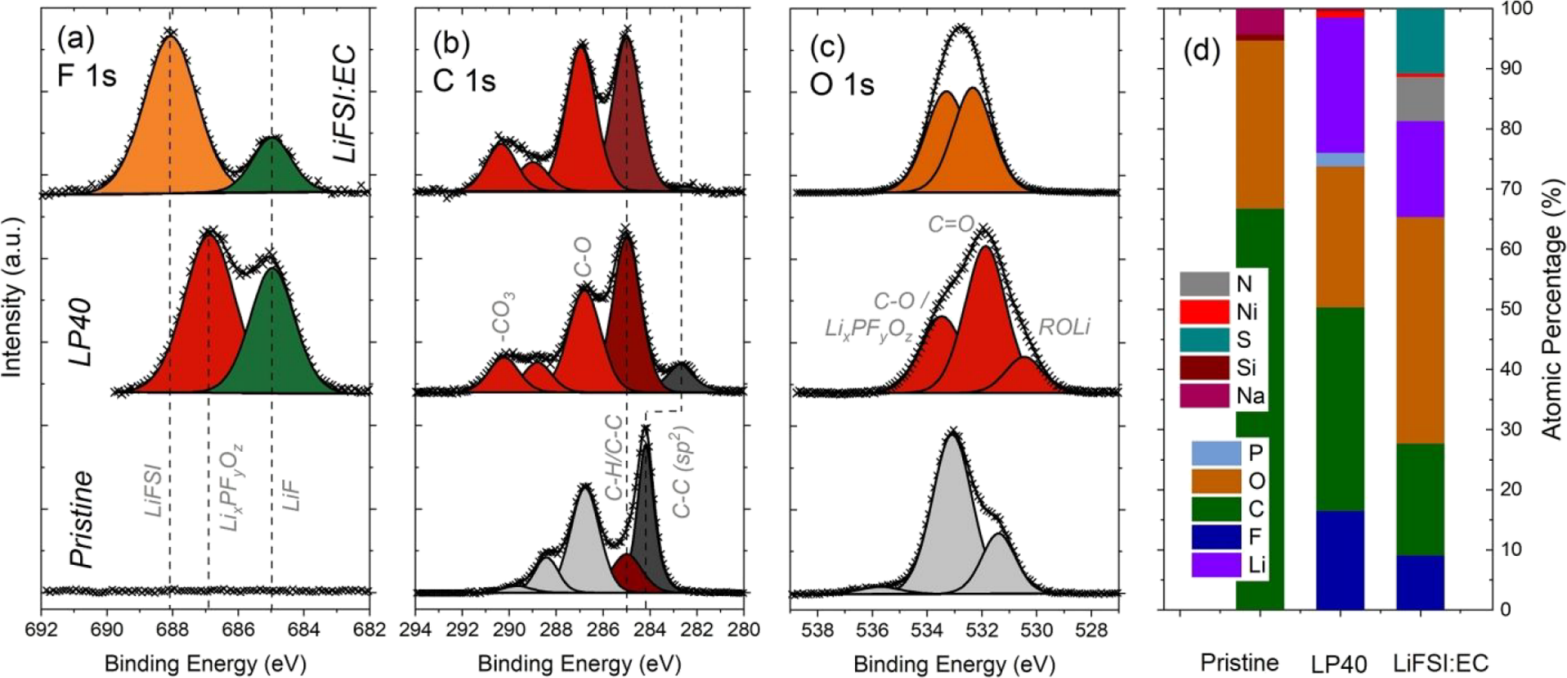
Normalized F 1s (a), C 1s (b), and O 1s (c) XPS spectra of Si/graphite electrodes before cycling (pristine) and after 10 cycles (using LP40 or 1:2 LiFSI:EC electrolytes). (d) Atomic percentage of the elements present in the analyzed top surface volume.

In the case of Si/graphite electrodes, the formation of surface films, that is, SEI, is easily observed in SEM images, in contrast to the NMC11 electrodes (see Figure 7). After cycling, active material and conductive additive particles lose their sharpness at the edges, and the gaps between particles seem to be filled with side reaction products. This is more significant in the case of the LiFSI-EC 1:2 electrolyte, as the primary particles become hard to detect because of more extensive SEI formation. In order to investigate the composition and surface film thickness further, XPS analysis was performed also on these electrodes, and the results are shown in Figures 8 and S10. In the F 1 s spectra, species related to LiPF_6_ and LiFSI salts are observed at 686.8 and 688.1 eV, respectively. Density functional theory (DFT) calculations have shown that the S-F bonds of LiFSI tend to break in the presence of Li in the nearby environment, and the formation of LiF is thermodynamically favorable.^48^ As expected, LiF is observed for both of the samples, while its concentration is significantly higher in the case of the LP40 electrolyte (also note the elemental concentration trends in Figure 8d).

In the C 1s spectra of the pristine Si/graphite electrode, active (graphite) and inactive electrode components (i.e., conductive carbon and binder) dominate the spectra, and their intensity decreases significantly after cycling. Before cycling, the graphite/conductive carbon peak (shown with dark gray in Figure 8) is located at 284.2 eV; however, this peak shifts to lower energy (282.7 eV) after 10 cycles, as also reported in earlier studies.^47,49^ Such a shift can be explained by differential charging effects^46^ because the electrically conductive bulk components would require a different calibration point as compared to SEI components. The intensity of this peak is negligible for the LiFSI:EC 1:2 electrolyte, indicating a thicker SEI formation, which is not surprising, following the SEM observations shown in Figure 7. As can be seen in Figure S10, binder-related Na 1s peaks also show a similar trend. A weak Na 1s peak is still visible for the LP40 electrolyte despite the lower kinetic energy of electrons (∼416 eV) and thus a lower XPS probing depth^50,51^ for the Na 1s binding energy of ∼1072 eV. For Si 2p (see Figure S10), no peaks are visible for both of the electrolytes. This is expected considering the high volume change of particles during lithiation/delithiation and thus a greater SEI breakdown/repair process on the Si particles.

The O 1s spectra of the pristine Si/graphite electrode (see Figure 8c) are mainly dominated by peaks related to binder (e.g., CMC) components and possibly to some degree by silicon surface oxides (e.g., SiO_2_) as well as adsorbed surface species (shown in light gray). Upon cycling, oxygenated species (shown with peaks in light-red color) are formed on the surface. Such species have a large overlap in binding energies in a narrow region, and it is unfortunately difficult to make a precise assignment of different species.^47^ In the case of the LP40 electrolyte, the spectra can be fitted with three main peaks: C–O-related species and Li_*x*_PF_*y*_O_*z*_ at ∼533.4 eV, C=O-related species (such as carbonates and carboxylates) at ∼531.9 eV, and lithium alkoxides at ∼530.5 eV. The elemental concentration of oxygen in the SEI layer increases significantly when the LiFSI:EC 1:2 electrolyte is used instead of the LP40 electrolyte (see Figure 8d). Overall elemental concentration trends indicate that the spectrum is mainly dominated by LiFSI-related species (e.g., S=O contributions).

It is well known that oxidation reaction products and transition metals dissolved from the cathode active material may migrate to the anode and be involved in side reactions and SEI formation (or penetrate through the existing SEI and damage its passivation ability).^3,7,52−54^ In this context, Figures 8d and S10 show that Ni can easily be detected on the surface of the Si/graphite electrode cycled with the LP40 electrolyte, while its concentration is visibly lower for the LiFSI:EC electrolyte. It can be speculated that this HC electrolyte either mitigates the transition-metal dissolution from the cathode or helps the formation of a more robust SEI on the Si/graphite electrode, which is then more resistant to transition-metal incorporation and damage. Nevertheless, it is not possible to draw firm conclusions from this observation only, considering that the measurements were performed with a fixed incoming photon energy (i.e., Al-Kα radiation, 1486.6 eV).

In summary, ex situ surface characterization of NMC111 and Si/graphite electrodes revealed distinct differences in surface film formation trends of the investigated electrolytes for both electrodes. In the case of NMC111 electrodes, the thickness of surface film deposits (both on NMC111 and carbon black network) was relatively higher for the LiFSI:EC electrolyte. Similar trends were also observed on Si/graphite electrodes in which the LiFSI:EC electrolyte resulted in thicker SEI formation consisting of LiFSI salt-related species, while also showing a lower concentration of lithium fluorides and nickel near the surface.

## Conclusions

In this study, the performances of highly concentrated LiFSI:EC electrolytes have been tested with respect to their low- and high-voltage stability using electrodes of Li metal, silicon/graphite, and NMC111 and LNMO electrodes. Generally, it is shown that this HCE constitutes a comparatively well-performing electrolyte system. The compatibility of the electrolyte with respect to Li metal is dependent on the salt concentration; LiFSI:EC 1:4 gives the optimum performance of the investigated salt loadings, while higher concentrations led to a negative impact on wettability (of the separator and/or the Li-metal surface). Such a negative effect is not seen with Si/graphite anodes, and it is possible to use electrolytes with higher salt concentrations in full cells. At high concentrations, the LiFSI-based electrolyte is shown to be stable against the corrosion of the Al current collector (up to ∼5.2 vs Li^+^/Li) and can be used with NMC111 electrodes without the addition of electrolyte additives. In NMC111–Si/graphite full cells, the LiFSI:EC electrolyte outperforms the conventional LP40 and LiTFSI-EC electrolyte during electrochemical cycling at practical currents (1 mA–2 mA cm^–2^). It also improves the self-discharge resistance in the charged state.

Surface characterization with SEM and XPS shows that thicker surface films are deposited on both NMC111 and Si/graphite electrodes after cycling (10 cycles) with the LiFSI-based electrolyte as compared to LP40. This difference is more pronounced on Si/graphite electrodes, and the results show that the LiFSI promotes the formation of a thicker SEI film with an outer part that is rich in oxygenated species, but relatively deficient in fluorinated components (e.g., LiF) and cathode-originating Ni ions. This suggests that such surface films are beneficial to cycling stability during longer-term cycling (>40 cycles) because the LiFSI-based electrolyte improved the capacity retention behavior considerably in both full- and half-cells. Further engineering of this simple electrolyte system could possibly enable more advanced electrolytes that are suitable for a wide range of applications, especially if the cost factor is mitigated with approaches such as dilution, for example, “locally concentrated electrolytes”, or using them in small amounts as interlayer electrolytes (e.g., between the electrode and a solid electrolyte) in hybrid-electrolyte cells.

## Supporting Information Available

Dynamic viscosity and differential scanning calorimetry results of LiTFSI:EC 1:6 and LiFSI:EC electrolytes; linear sweep voltammetry (LSV) results of LiFSI:EC 1:2-based electrolytes in Al-Li cell configuration; ionic conductivities of LiFSI:EC electrolytes at different salt concentrations; electrochemical cycling results of Si/graphite–Li half cells; electrochemical cycling results of NMC111–Li half cells cycled with a high-precision charger at 55 °C; Coulombic efficiency (CE) measurements using the high-precision charger; electrochemical testing of high-voltage LiNi_0.44_Mn_1.56_O_4_-based full cells with Si/graphite electrodes; SEM images of NMC111 electrodes before and after 10 cycles; XPS results of NMC111 electrodes before and after 10 cycles; and XPS results of Si/graphite electrodes before and after 10 cycles (PDF)
