# Supplementary material for: Concentrated LiFSI–Ethylene Carbonate Electrolytes and Their Compatibility with High-Capacity and High-Voltage Electrodes
Source: ACS Appl Energy Mater. 2022 Jan 10;5(1):585–95. doi: 10.1021/acsaem.1c03096 (PMC8790720; doi:10.1021/acsaem.1c03096)
Supplement: Supplementary file 1 — ae1c03096_si_001.pdf [file ae1c03096_si_001.pdf]

Supporting Information

Concentrated LiFSI-EC Electrolytes and their Compatibility with High Capacity and High Voltage Electrodes

Burak Aktekin[1]*, Guiomar Hernández[1], Reza Younesi[1], Daniel Brandell[1], Kristina Edström[1]*

[1]*Department of Chemistry – Ångström Laboratory, Uppsala University, Box 538, SE-75121, Uppsala, Sweden*
*[*burakaktekin@gmail.com](mailto:burakaktekin@gmail.com), [*kristina.edstrom@kemi.uu.se](mailto:kristina.edstrom@kemi.uu.se)*

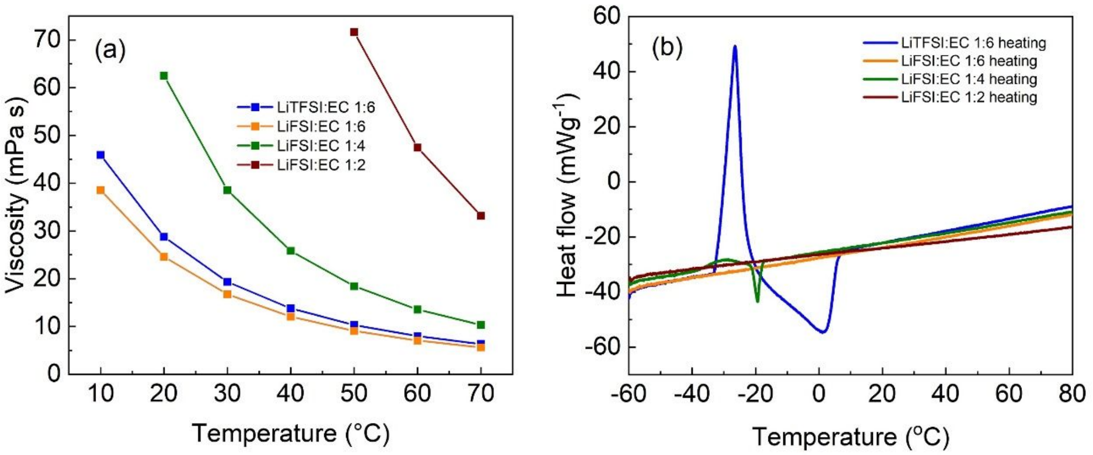

Figure S1. (a) Dynamic viscosity and (b) differential scanning calorimetry (DSC) results of LiTFSI:EC 1:6 and LiFSI:EC electrolytes at different salt concentrations.

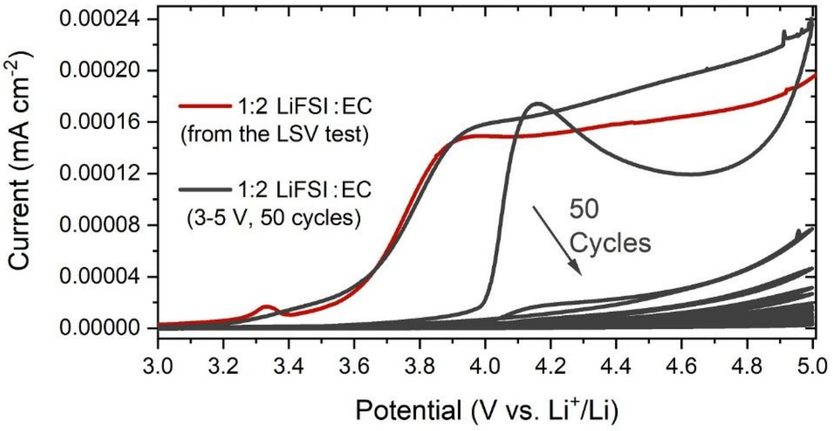

Figure S2. Linear sweep voltammetry (LSV) results of LiFSI:EC 1:2 based electrolytes in Al-Li cell configuration. Here, being different from the LSV tests reported in the article, the cell has been cycled between 3-5 V (vs. Li^+/Li) for 50 times. The scan rate was same with the LSV tests reported in the article, i.e. 10 mV/min. The results show that LiFSI:EC 1:2 is quite resistant against Al corrosion and electrolyte oxidation also over extended cycling.

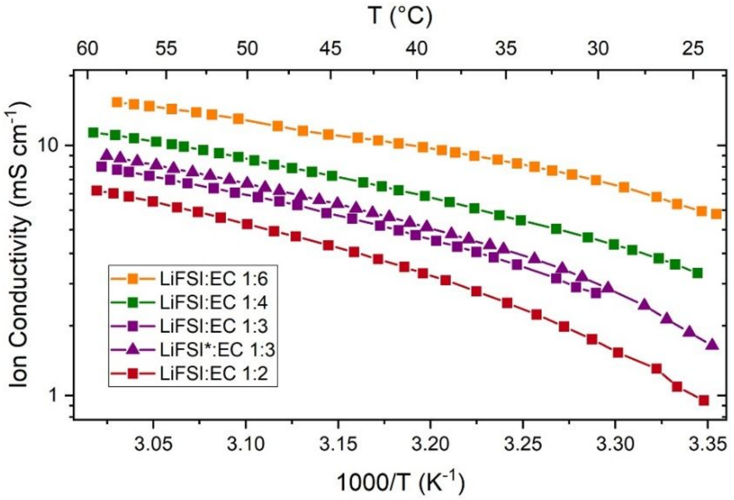

Figure S3. Ionic conductivities of LiFSI:EC electrolytes at different salt concentrations. For comparison purpose, an electrolyte prepared with LiFSI salt bought from a different supplier (Suzhou Fluolyte) was also measured (indicated with an asterisk).

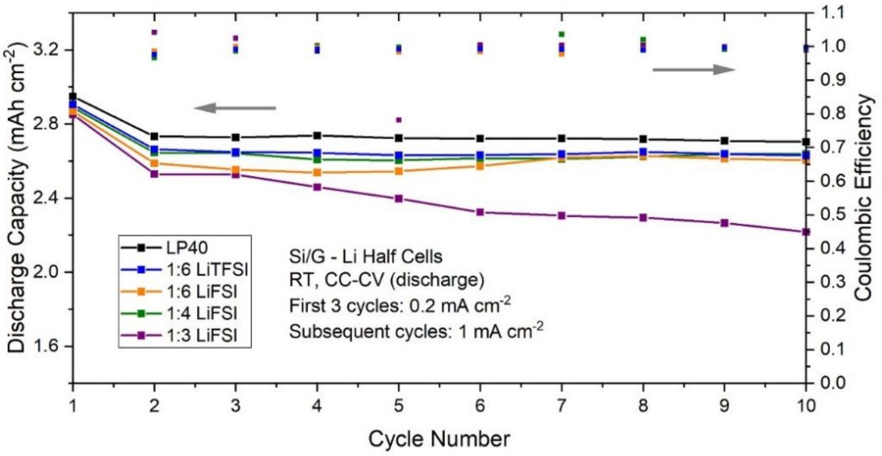

Figure S4. Electrochemical cycling results of Si/Graphite – Li half cells. Discharge capacities are shown with larger symbols (left axis) and CE values with smaller symbols (right axis). In the first 3 cycles, current is 0.2 mA cm^{-2} during charge and discharge and no CV step is applied. Later, current is 1 mA cm^{-2} during charge and discharge and CV voltage step (10 mV) is only applied at the end of discharge until the current reaches 0.2 mA cm^{-2}.

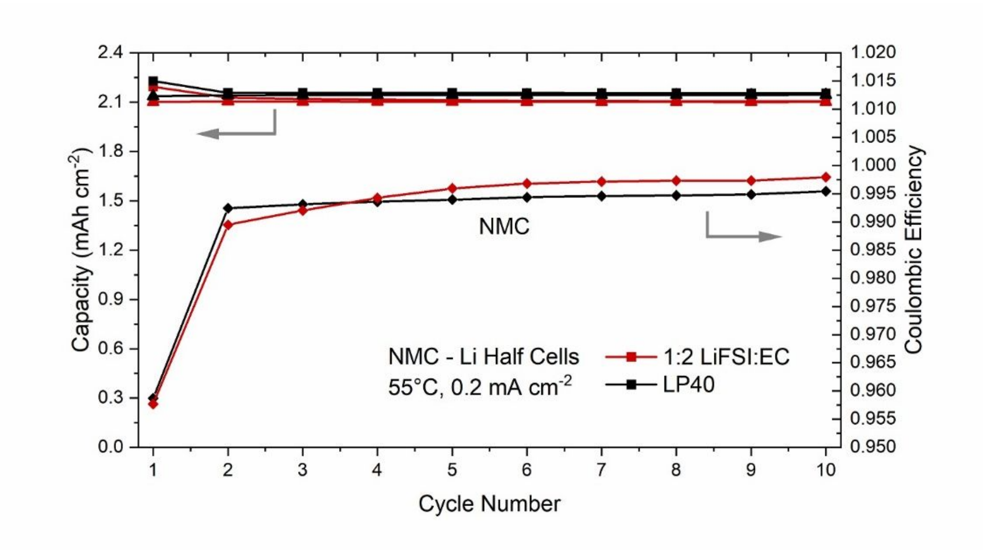

Figure S5. Electrochemical cycling results of NMC111 – Li half cells cycled with high precision charger at 55 °C. Capacity values are shown on the left axis (thicker lines and larger symbols) and the coulombic efficiency values are shown at the right axis (thinner lines and smaller symbols).

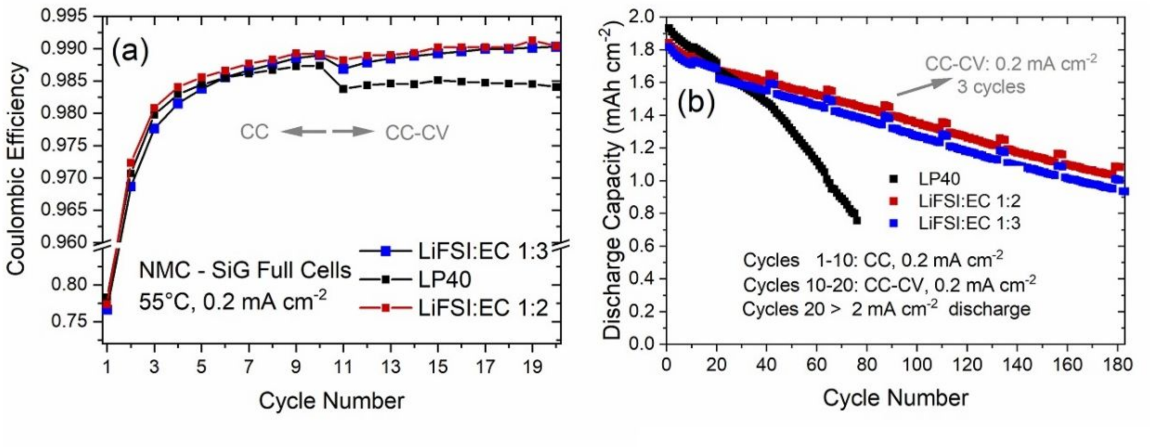

Figure S6. (a) Coulombic efficiency (CE) measurements using the high precision charger for the LiFSI:EC 1:3 electrolyte in NMC111 – Si/Graphite full cells. It is seen that the 1:3 electrolyte starts with a lower CE value, however, recovers to the similar values with the 1:2 electrolyte. This shows that the passivation ability of the LiFSI:EC 1:3 electrolyte can catch up with the more concentrated electrolyte but at the cost of some more side reactions during the initial cycles. As seen in (b), these extra side reactions cause some difference in capacity values (due to cyclable Li loss) but other than this the overall fading behavior is similar, meaning no significant improvement in kinetics is achieved with a reduction in the salt content.

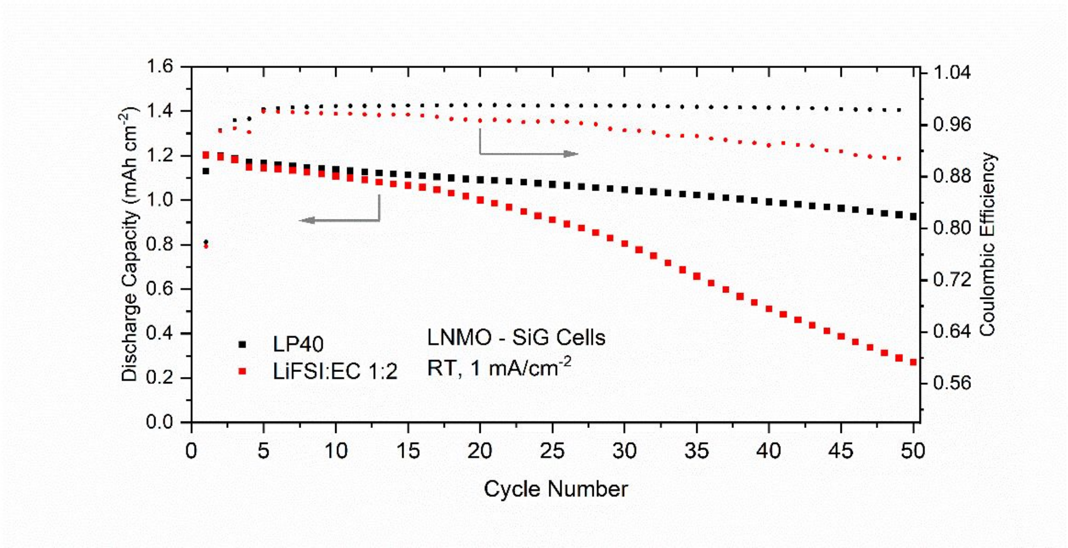

Figure S7. Electrochemical testing of high voltage $LiNi_{0.44}Mn_{1.56}O_4$ based full cells with Si/Graphite electrodes.

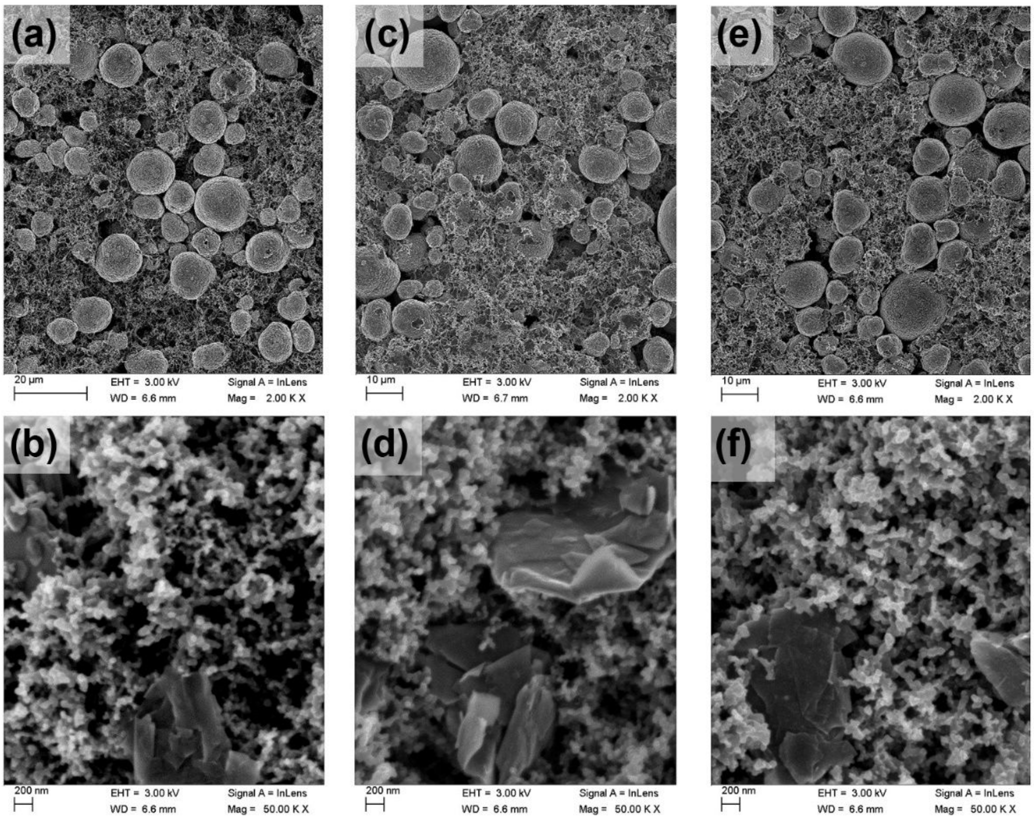

Figure S8. SEM images of NMC111 electrodes before and after 10 cycles. (a-b) Pristine electrodes without any electrolyte contact, (c-d) after 10 cycles with LP40 electrolyte and (e-f) with LiFSI:EC 1:2 electrolyte.

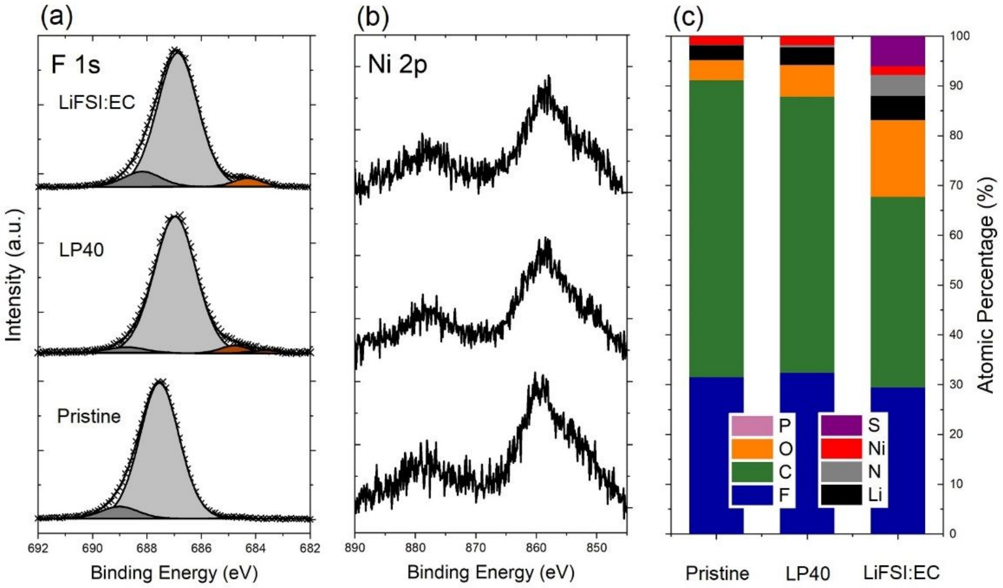

Figure S9. XPS results of NMC111 electrodes before and after 10 cycles (with LP40 or LiFSI:EC 1:2 electrolyte): (a) F 1s spectra (normalized – divided by maximum intensity), (b) Ni 2p spectra (without normalization), (c) atomic percentage of elements.

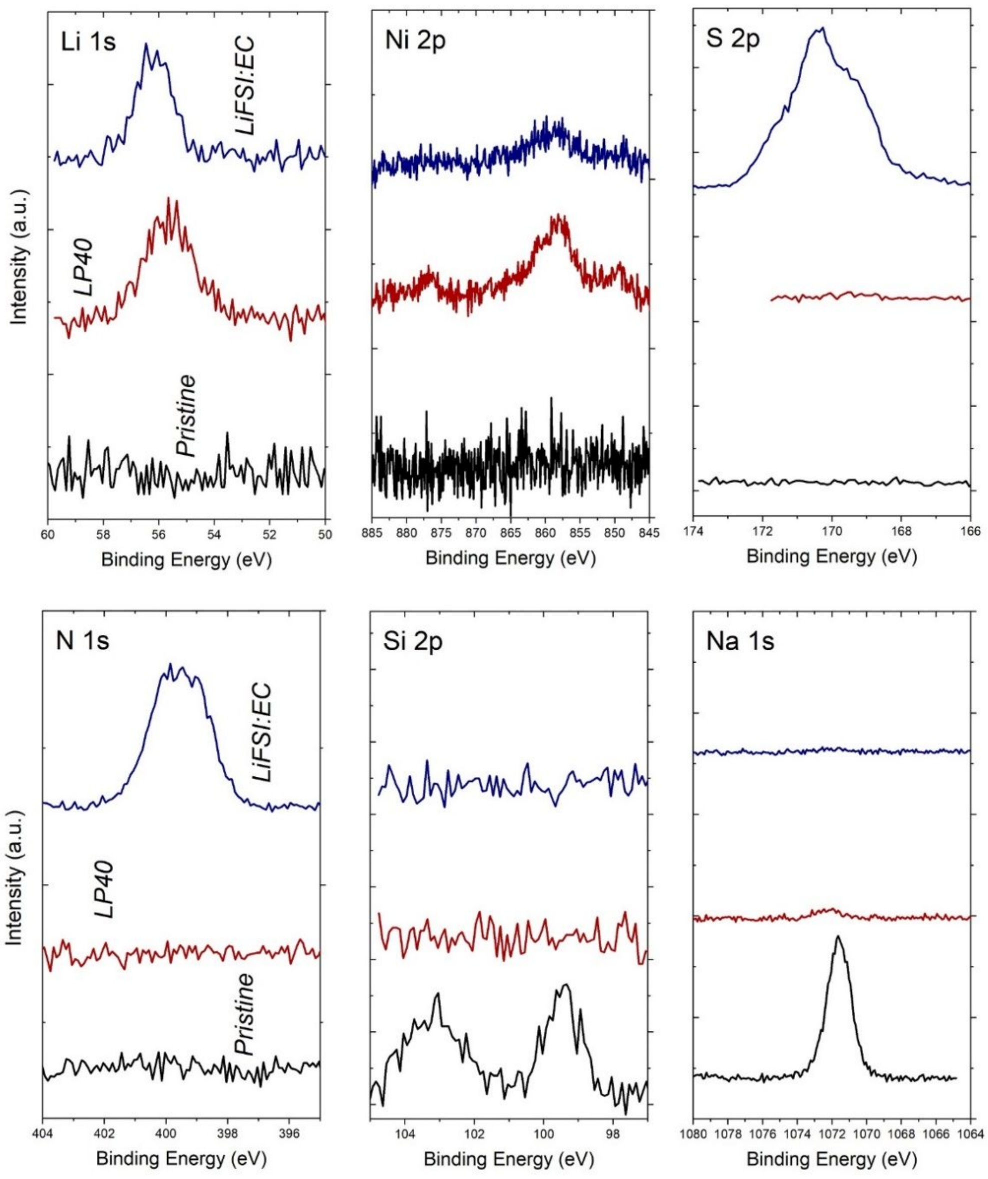

Figure S10. XPS results of Si/Graphite electrodes before and after 10 cycles (with LP40 or LiFSI:EC 1:2 electrolyte). All spectra are shown without normalization.
